# DTG + 3TC dual therapy for the treatment Naïve patients with viral load exceeding 500,000 copies/mL: a retrospective study

**DOI:** 10.1186/s12879-024-09624-2

**Published:** 2024-07-22

**Authors:** Yanyun Dou, Guangfu Liao, Ruichao Lu, Lingsong Su, Ke Lan, Zhihao Meng, Shanfang Qin, Wei Huang, Yuanlong Xu, Yu Lv, Yuhong Wen, Shuanglai Lan, Yong Zuo, Yong Zhang

**Affiliations:** Chest Hospital of Guangxi Zhuang Autonomous Region, Liuzhou, Guangxi Province China

**Keywords:** Dolutegravir, Lamivudine, Dual therapy, HIV

## Abstract

**Background:**

Antiretroviral therapy (ART) has transformed HIV management, with various regimens available. Dolutegravir (DTG) plus lamivudine (3TC) dual therapy is now the one of the first line regimens.

**Methods:**

A retrospective, observational study included treatment naïve people living with HIV (PLWH) with baseline HIV RNA viral load (VL) greater than 500,000 copies/mL from March 2020 to June 2022. PLWH on DTG + 3TC were included in the 2DR group, while others on INSTI-based three-drug regimens were divided in the 3DR group. Viral suppression, immunological recovery, and safety were assessed.

**Results:**

The study included 52 PLWH, with no significant baseline differences. Virologic suppression rates at weeks 24 and 48 were similar in both groups, even with baseline HIV RNA VL greater than 1,000,000 copies/mL. CD4 + T cell counts improved rapidly. No serious adverse effects were reported.

**Conclusions:**

DTG + 3TC dual therapy demonstrates effectiveness in treatment naïve PLWH with high baseline HIV RNA VL, suggesting its potential as a first line regimen for all treatment naïve PLWH.

## Background

With the development and widespread adoption of antiretroviral therapy (ART), HIV infection has transformed from a lethal disease to a manageable condition. People living with HIV (PLWH) now have a life expectancy comparable to the general population [1]. Standard ART initiation regimens typically include two nucleoside reverse transcriptase inhibitors (NRTIs) and a third core agent [2–5]. The second-generation Integrase strand transfer inhibitor (INSTI) has emerged as the preferred core agent due to its high resistance barrier, high efficacy, optimal safety profile, and reduced drug-drug interactions(DDIs).

To reduce long-term toxicities and mitigate side effects, two-drug regimens (2DRs) have become a new focus in HIV management. Recent years have seen increasing investigations in the effectiveness of 2DRs for HIV management. Notably, DTG + 3TC is the only dual therapy currently recommended by international guidelines as a first-line regimen for treatment naïve PLWH [3–5]. This recommendation stems from the GEMINI-1 and 2 phase III clinical trials, which demonstrated the long-term non-inferior efficacy of DTG + 3TC compared to DTG + TDF/FTC in treatment naïve PLWH [6, 7]. However, DTG + 3TC is advised only for treatment naïve PLWH with a baseline HIV RNA viral load (VL) lower than 500,000 copies/mL, a limitation not seen in other INSTI-based three-drug regimens (3DRs) [3–5].

In China, where the distribution of resources is highly uneven, baseline HIV RNA VL and resistance tests are not readily available in many regions, primarily due to their high costs or accessibility. This presents a challenge in HIV management. Additionally, the practice of rapid start is common, and some hospitals may experience delays in obtaining test results. Consequently, many doctors initiate ART without baseline HIV RNA VL testing and genotype testing. A significant proportion of HIV diagnoses in China are delayed or are aging population. As a result, these PLWH are frequently associated with DDI and organ dysfunction, potentially making DTG + 3TC dual therapy a more suitable initial treatment [8, 9].

Although DTG + 3TC is approved for use in both treatment naïve and experienced PLWH without HIV RNA VL restrictions and is widely used in naïve PLWH in China due to its high efficacy, resistance barrier, fewer DDIs, and lower cost, the Chinese guideline still recommends initiating DTG + 3TC in naïve PLWH with a baseline HIV RNA VL lower than 500,000 copies/mL. Thus, our study aims to evaluate retrospectively the effectiveness of DTG + 3TC dual therapy in treatment naïve PLWH with baseline HIV RNA VL greater than 500,000 copies/mL.

## Materials and methods

We conducted a single-center retrospective, observational study at the Chest Hospital of Guangxi Zhuang Autonomous Region, a facility managing over 10,000 PLWH. The study, approved by the Ethical Committee (NO.20,201,005). The inclusion criteria consisted of newly diagnosed PLWH with age 18 years or over, with baseline HIV RNA VL greater than 500,000 copies/mL. And the PLWH should initiate with INSTI based regimen. The data regarding clinical information and laboratory tests were obtained from the review of medical records. PLWH on DTG + 3TC (either as separate pills or as a single-tablet regimen) were included in the 2DR group, while those on other INSTI-based 3DRs were included in the 3DR group. PLWH missing either 6-month or 12-month follow-up data were excluded. Data were sourced from medical health records and compiled into an anonymized Excel^®^ dataset.

The study collected demographic information (age and gender), HIV-specific characteristics (CD4 + T cell count, HIV-1 RNA, and AIDS-related opportunistic infections), and other vital blood tests, such as lipid profile, renal function, and liver function. HIV diagnosis and AIDS were defined according to World Health Organization (WHO) guidelines. Viral suppression was defined as an undetectable viral load or lower than 50 copies/mL, and virological failure (VF) as a detectable viral load above 1000 copies/mL after a minimum of 6 months of ART [2]. Serious adverse effects (SAE) were also included in the study.

Statistical analysis was performed using SPSS Statistics for Windows, version 26.0 (IBM, Armonk, NY, USA). Demographics were summarized using descriptive statistics, with categorical variables reported as absolute numbers (proportion). Con-tinuous variables were expressed as mean and standard deviation (SD) for normally distributed data, and median with interquartile range (IQR) for non-normally distributed data. Comparison between the 2DR and 3DR groups was conducted using the χ2 test for categorical variables and the unpaired T-test (for normally distributed data) or the Mann–Whitney U test (for non-normally distributed data) for continuous variables. Intra-subject median changes from baseline to follow-up were analyzed using the paired T-test or the paired Wilcoxon test, excluding PLWH classified as discontinuations. A p-value of less than 0.05 was considered statistically significant.

## Results

**Fig. 1:**
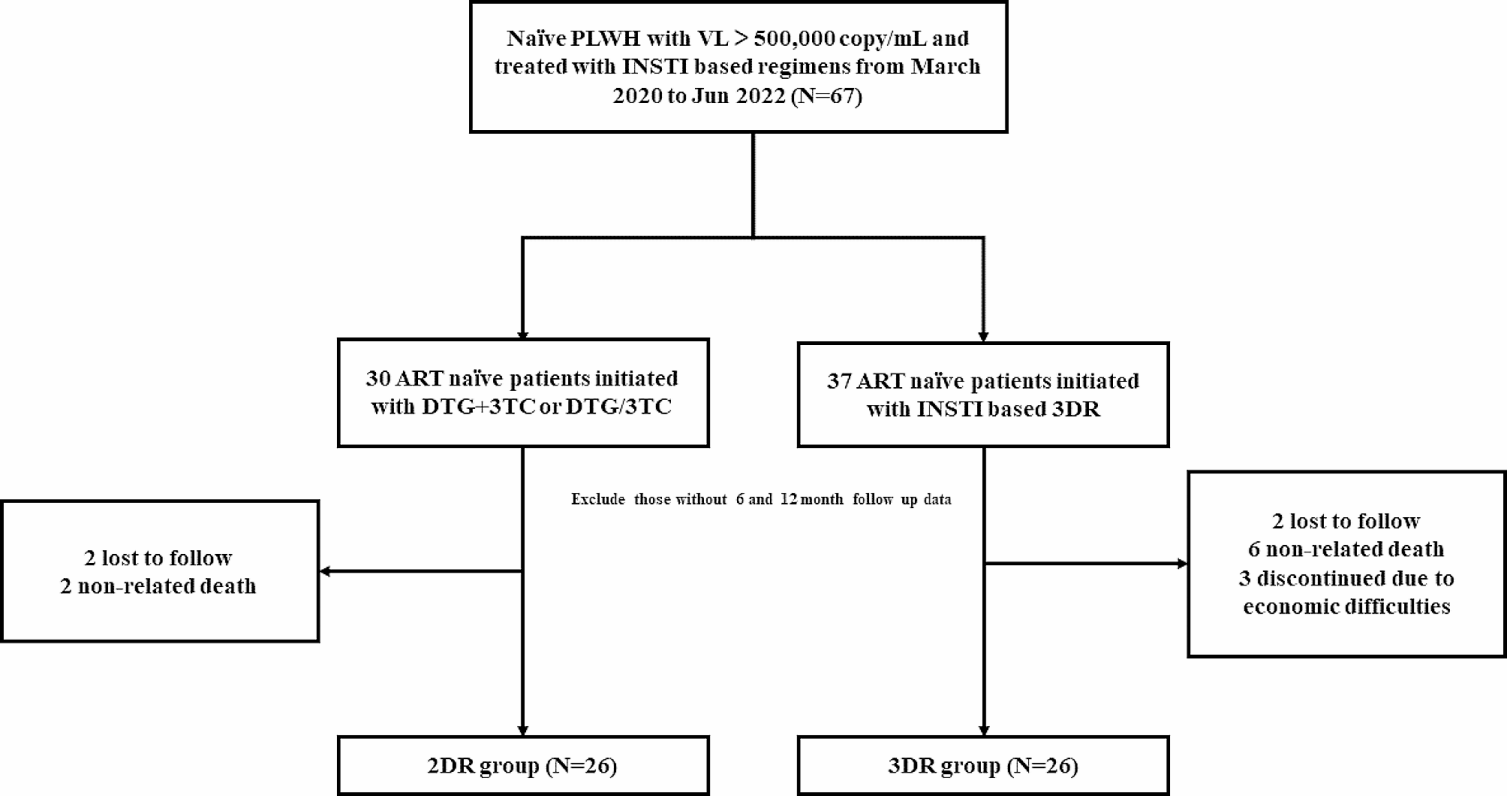
Flow chart for the participant selection method used in this study

The patient selection process, as depicted in Fig. 1, involved evaluating 67 treatment naïve PLWH with baseline HIV RNA VL greater than 500,000 copies/mL who began treatment with INSTI-based regimens. Of these, 15 PLWH were excluded, resulting in 26 individuals in the 2DR group and 26 in the 3DR group. There were 2 and 6 deaths in the 2DR and 3DR groups, respectively, none of which were treatment related.

The baseline characteristics of the 52 PLWH, as presented in Table 1, showed that the majority in both groups were male, with a median age of 59.5 years vs. 56 years in 2DR and 3DR group, respectively. Specifically, 84.6% in the 2DR group and 57.7% in the 3DR group were over 50 years old. In both groups, a high percentage of PLWH had CD4 cell counts below 200 cells/µL, with median counts of 55 in the 2DR group and 50 cells/µL, in the 3DR group, respectively. Over half of the PLWH in each group had baseline HIV RNA VL greater than 1,000,000 copies/mL. AIDS-related opportunistic infections were present in 65.4% of the 2DR group and 69.2% of the 3DR group. There were no significant differences in lipid profiles, renal function, and liver function between the two groups. In the 3DR group, 42.3% initiated treatment with first-generation INSTI-based 3DRs, and 26.9% with BIC/TAF/FTC.

**Table 1:** Baseline characteristics of PLWH included in this study

Characteristic	2DR group(*N* = 26)	3DR group(*N* = 26)	*P* value
Gender, male, *n* (%)	18(69.2)	19(73.1)	0.760
Age, years, median (IQR)	59.5(53.8,69.3)	56.0(43.8,69.8)	0.301
Age >50 years, *n* (%)	22(84.6)	15(57.7)	0.032
CD4 + cell count			
Median (IQR), cell/µL	55.0(25.3,198.8)	50.0(25.8,154.3)	0.481
< 200 cell/µL, *n* (%)	20(76.9)	22(84.6)	0.482
HIV viral load			
Median (IQR), copies/mL	1,080,720.0(649,624.5,2,261,758.8)	1,115,434.0(674,327.8,1,830,525.3)	0.927
>1,000,000 copies/mL, *n* (%)	15(57.7)	14(53.8)	0.780
AIDS-related opportunistic infections, *n* (%)	17(65.4)	18(69.2)	0.768
Lipid profile			
Triglyceride, mmol/L, median (IQR)	1.3(1.0,2.1)	1.4(1.1,2.4)	0.410
LDL, mmol/L, median (IQR)	2.5(1.9,2.9)	2.4(2.0,3.1)	1.000
Cholesterol, mmol/L, median (IQR)	3.8(3.4,4.4)	3.8(3.2,4.6)	0.993
Renal function			
UREA, mmol/L, median (IQR)	4.1(2.6,4.7)	3.7(2.6,4.4)	0.798
Creatinine, umol/L, median (IQR)	67.0(51.5,82.8)	63.5(47.0,85.3)	0.749
CKD-EPI, ml/min/1.73m^2^, median (IQR)	95.2(84.5,103.9)	106.7(77.2,118.9)	0.453
Liver function			
AST, IU/L, median (IQR)	26.5(21.8,48.5)	37.0(24.0,45.5)	0.522
ALT, IU/L, median (IQR)	22.0(15.5,42.5)	24.5(18.8,31.8)	0.812
Total bilirubin, µmol/L, median (IQR)	6.4(5.0.10.2)	6.5(4.3,8.2)	0.596
ART Regimen, *n* (%)			
DTG + 3TC	26(100)		NA
TDF + 3TC + DTG		7(26.9)	
AZT + 3TC + DTG		1(3.8)	
EVG/c/TAF/FTC		11(42.3)	
BIC/TAF/FTC		7(26.9)	

**Fig. 2:**
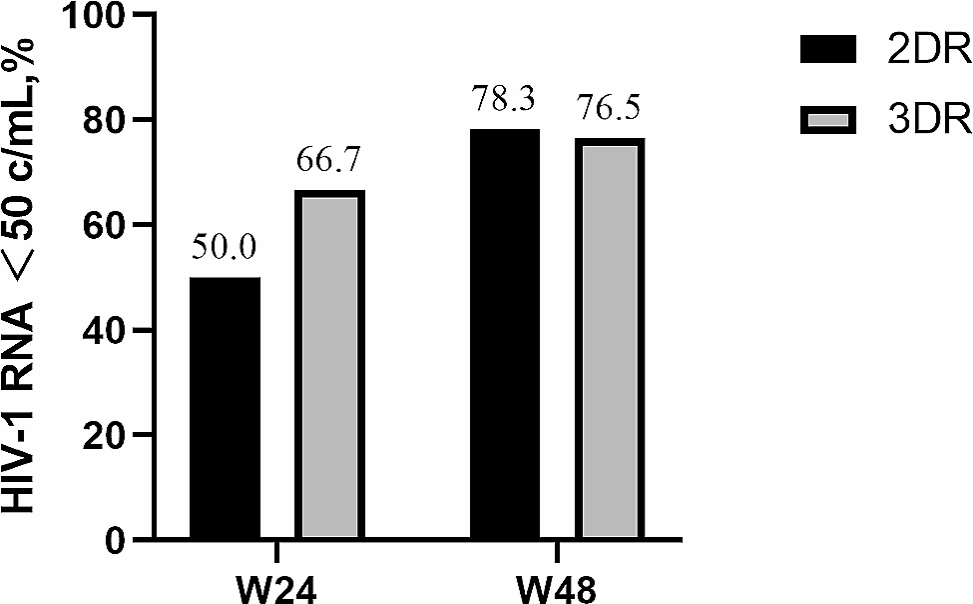
Proportion (%) of HIV RNA < 50 copies/mL and HIV RNA < 200 copies/mL in 2DR and 3DR group at Week 24 and Week 48 follow-up visit

PLWH had only 6-month or 12-month follow-up data were also included in this study. Figure 2 shows that the virologic suppression rate in the 2DR group was 50.0% (12/24) at week 24 and 78.3% (18/23) at week 48. In the 3DR group, the rates were 66.7% (14/21) at week 24 and 76.5% (13/17) at week 48. Most PLWH in both groups achieved HIV RNA VL under 200 copies/mL at weeks 24 and 48, indicating no significant difference in virus reduction between the two groups. There was one instance of VF in the 3DR group, with an HIV RNA VL of 43,161 copies/mL at week 48, and no follow-up visit at week 24. Among those with baseline HIV RNA VL greater than 1,000,000 copies/mL, 85.7% (12/14) in the 2DR group and 70.0% (7/10) in the 3DR group achieved virologic suppression at week 48.

**Fig. 3:**
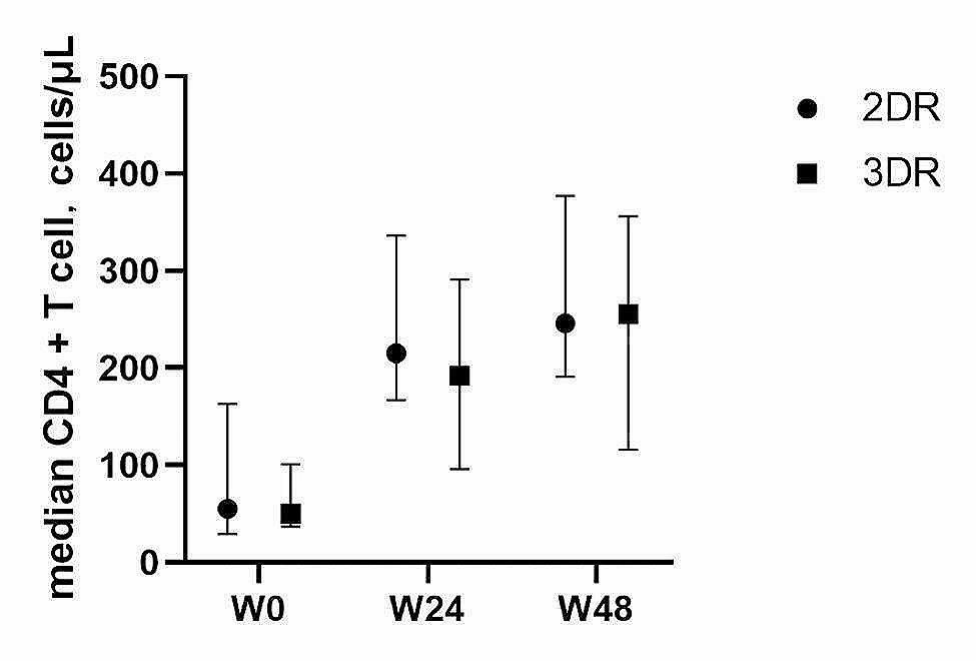
Changes of median CD4 + T cell count in 2DR and 3DR group at 6- and 12-month follow-up visit

The median CD4 + T cell count in the 2DR group increased from 55.0 cells/µL (IQR: 25.3, 198.8) at baseline to 215.0 cells/µL (IQR: 157.0, 379.0) after 24 weeks and to 246.0 cells/µL (IQR: 180.0, 404.0) after 48 weeks. In the 3DR group, the count rose from 50.0 cells/µL (IQR: 25.8, 154.3) at baseline to 192.0 cells/µL (IQR: 95.5, 301.3) after 24 weeks and to 255.5 cells/µL (IQR: 117.3, 352.8) after 48 weeks (Fig. 3) on the treatment.

As per Table 2, lipid profiles, including triglycerides (TG), low-density lipoprotein cholesterol (LDL-C), and total cholesterol (TC), were evaluated. Post-treatment, there was no significant change except for increased LDL-C levels in the 2DR group and cholesterol in both groups. Improved renal function was observed in both groups, indicated by changes in blood urea nitrogen (BUN), creatinine, and estimated glomerular filtration rate (eGFR). Liver enzymes, such as aspartate aminotransferase (AST) and alanine aminotransferase (ALT), significantly improved in both groups, while total bilirubin notably increased in the 2DR group. The study did not include any SAE.

**Table 2:** Laboratory parameters at between baseline, Week 24 and Week 48 in the 2DR and 3DR groups

median (IQR)	2DR group					3DR group				
	W0	W24	*P*	W48	*P*	W0	W24	*P*	W48	*P*
Triglyceride, mmol/L	1.3 (1.0,2.1)	1.5 (1.1,2.0)	0.484	1.6 (1.2,2.1)	0.176	1.4 (1.1,2.4)	1.6 (1.2,2.31	0.563	1.8 (1.4,3.8)	0.116
LDL, mmol/L	2.5 (1.9,2.9)	2.6 (2.5,3.7)	0.003	3.0 (2.4,3.5)	0.022	2.4 (2.0,3.1)	2.8 (2.0,3.8)	0.064	3.1 (2.4,4.0)	0.055
Cholesterol, mmol/L	3.8 (3.4,4.4)	4.6 (3.7,5.5)	0.001	5.0 (4.2,6.2)	<0.001	3.8 (3.2,4.6)	4.4 (3.6,5.3)	0.056	4.9 (4.1,5.8)	0.005
BUN, mmol/L	4.1 (2.6,4.7)	5.2 (3.8,7.0)	<0.001	5.8 (4.1,6.9)	<0.001	3.7 (2.6,4.4)	5.1 (3.9,5.6)	0.001	4.8 (3.7,6.0)	0.077
Creatinine, umol/L	67.0 (51.5,82.8)	81.0 (63.5,103.0)	<0.001	81.0 (70.0,99.0)	<0.001	63.5 (47.0,85.3)	85.0 (68.8,93.0)	0.001	75.0 (66.0,99.0)	0.001
CKD-EPI, ml/min/1.73m^2^	95.2 (84.5,103.9)	87.4 (66.8,99.7)	<0.001	88.0 (67.6,95.4)	<0.001	106.7 (77.2,118.9)	88.6 (66.9,106.4)	0.002	95.4 (59.3,108.4)	0.001
AST, IU/L	26.5 (21.8,48.5)	20.0 (19.0,25.0)	0.003	20.0 (17.0,26.0)	<0.001	37.0 (24.0,45.5)	23.5 (18.3,28.8)	0.001	26.0 (20.0,33.0)	0.046
ALT, IU/L	22.0 (15.5,42.5)	12.0 (9.0,15.5)	<0.001	12.0 (8.0,16.0)	0.003	24.5 (18.8,31.8)	15.5 (13.0,21.8)	<0.001	20.0 (13.0,28.0)	0.073
Total bilirubin, µmol/L	6.4 (5.0.10.2)	8.48 (7.0,13.0)	0.054	9.46 (7.18,11.49)	0.042	6.5 (4.3,8.2)	7.3 (5.9,11.0)	0.095	7.6 (5.1,9.6)	0.407

## Discussion

In this retrospective study, we observed similar viral suppression rates and substantial immunological recovery at week 48 in treatment naïve PLWH with a HIV RNA VL greater than 500,000 copies/mL treated with DTG + 3TC dual therapy, compared to those on INSTI-based 3DRs. No VF or SAE was noted in the DTG + 3TC group, aligning with findings from randomized clinical trials and real-world studies assessing DTG + 3TC efficacy and safety in treatment naïve PLWH [7, 10–17].

While guideline-recommended INSTI-based 3DRs and DTG + 3TC are generally the initial ART regimens for most treatment naïve PLWH due to their durable virologic efficacy, high resistance barrier, simplification of drug administration, and favorable toxicity profiles, DTG + 3TC is not yet advised for initiating treatment in treatment naïve PLWH with HIV RNA VL greater than 500,000 copies/mL [3–5]. Previous clinical studies have not extensively included such individuals. In the GEMINI 1 and 2 studies, among the 13 ART-naïve PLWH with baseline HIV RNA VL greater than 500,000 copies/mL, 84.6% achieved viral suppression at week 48 [7]. In the STAT study, ITT analysis found that 89% (17/19) naïve PLWH with baseline HIV RNA VL greater than 500,000 copies/mL achieved viral suppression at week 48 [17]. Similar high suppression rates were also noted in several real-world studies from China including individuals with HIV RNA VL greater than 500,000 c/ml. [11–14]. These findings suggest that DTG + 3TC dual therapy can effectively suppress high HIV RNA VL in naïve PLWH, even in those with HIV RNA VL greater than 1,000,000 copies/mL.

A key topic of discussion is the potency of DTG + 3TC dual therapy in achieving rapid initial HIV RNA VL decline. The rapid reduction in viral load, as supported by the percentage of PLWH who achieved viral suppression at week 4 (72% in the 2DR and 70% in the 3DR) and the rapid median time to viral suppression (29 days), demonstrated that the initial antiviral potency of DTG + 3TC is similar to that of DTG + TDF/3TC [6]. In the STAT study, 92% PLWH receiving DTG + 3TC achieved viral suppression, among the 19 PLWH with baseline HIV RNA VL greater than 500,000 copies/mL, 13 (68%) were suppressed at Week 24 [18]. However, a Chinese study noted a significantly lower viral suppression rate at week 24 in the high HIV RNA VL group [11]. Although no significant difference in HIV RNA reduction or viral sup-pression percentages was found in many studies [6, 10, 14, 15, 18], our study observed a lower viral suppression rate in PLWH with baseline HIV RNA VL greater than 500,000 copies/mL receiving DTG + 3TC dual therapy. However, it takes longer time to reach viral suppression in PLWH with higher baseline HIV RNA VL [19]. Despite this, it’s not yet clear if the initial HIV RNA VL decline of DTG + 3TC is inferior to 3DR in PLWH with high baseline HIV RNA VL, and more evidence is needed.

Virological and immunological indicators are crucial for evaluating the recovery and antiviral effectiveness in PLWH [20]. Our study demonstrated rapid increases in CD4 counts at weeks 24 and 48, indicating improved immune function after DTG + 3TC treatment, comparable to INSTI-based 3DRs in PLWH with high HIV RNA VL and low CD4 cell counts.

Regarding renal function, INSTIs are known to inhibit the organic cation trans-porter 2 in kidney proximal tubule cells, leading to increased serum creatinine levels without causing true renal impairment [21]. Both groups showed a significant increase in serum creatinine levels and changes in eGFR, common in clinical practice. Elevated LDL-C or TC levels, key risk factors for cardiovascular disease, were also observed post-treatment in PLWH initiating DTG + 3TC, in line with findings from the GEMINI 1 and 2 studies [6]. Lipid indicators are complex and influenced by many factors, including non-fasting states at follow-up visits, which might introduce some variability in our study.

This study has limitations, including its retrospective nature, small sample size, and reliance on routine laboratory data, which might introduce bias. The economic conditions in Guangxi Province might have affected the frequency of viral load testing and led to some PLWH not being enrolled or discontinuing treatment. Additionally, baseline drug resistance tests were not available for all PLWH, but our results align with a Spanish real-world study where naïve PLWH without baseline drug resistance test results still achieved high rates of viral suppression [15]. This may be due to the low incidence of pre-existing drug resistance to 3TC and the high resistance barrier of DTG [22].

Our study indicates that DTG + 3TC dual therapy is effective in PLWH with baseline HIV RNA VL greater than 500,000 copies/mL, showing no VF and significant immunological improvement. Hence, DTG + 3TC might also be a viable option for naïve PLWH with baseline HIV RNA VL greater than 500,000 copies/mL or without baseline HIV RNA VL results.

## Data Availability

The datasets used and/or analysed during the current study are available from the corresponding author on reasonable request.
